# Supplementary material for: Comparative study on the virulence of mycobacteriophages
Source: J Virol. 2025 May 21;99(6):e01920-24. doi: 10.1128/jvi.01920-24 (PMC12172466; doi:10.1128/jvi.01920-24)
Supplement: Supplemental figures — Figures S1 to S4. [file jvi.01920-24-s0001.pdf]

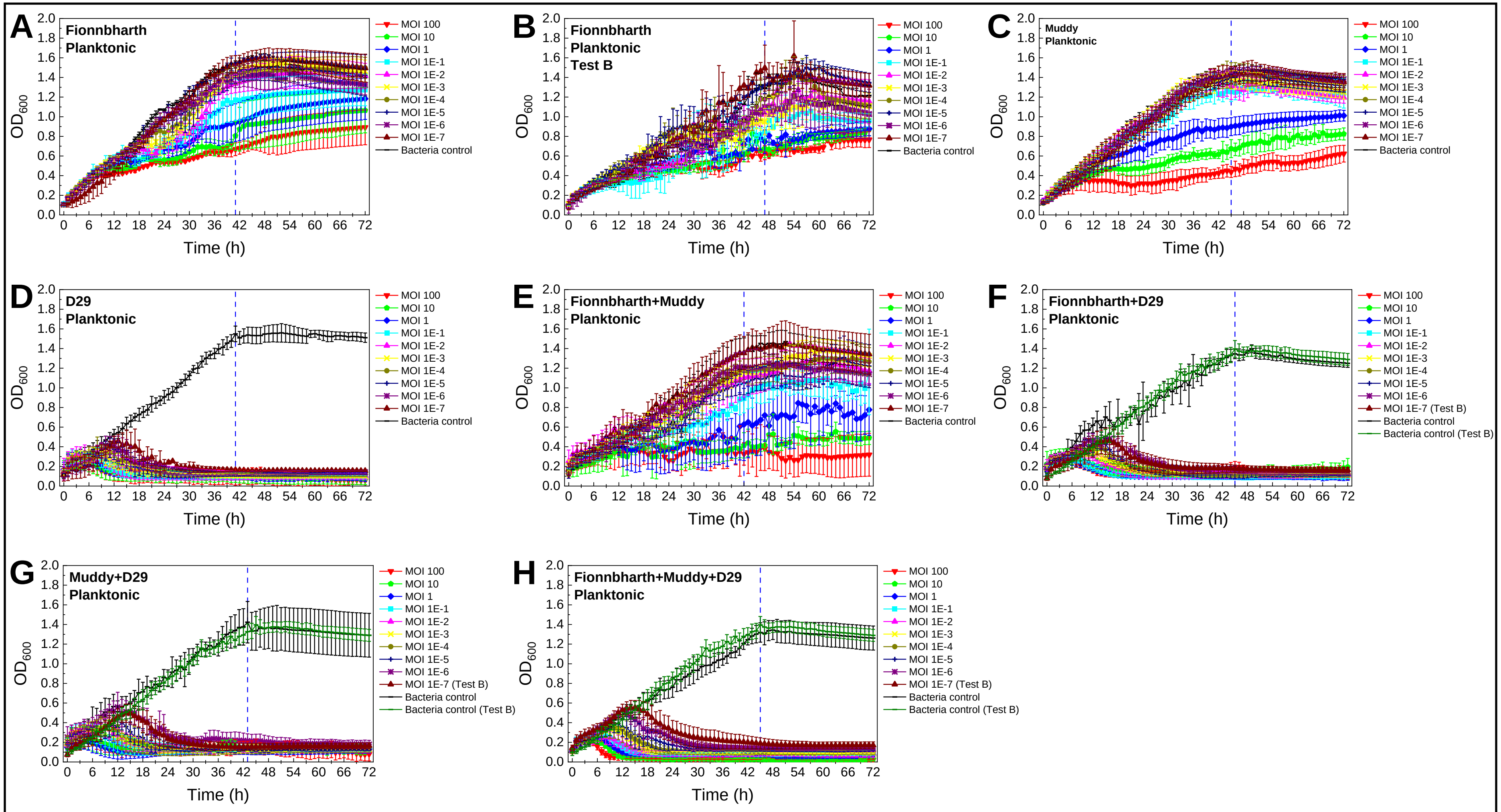

Supplementary Figure 1. Reduction curves for infection under planktonic growth. Reduction curves for phages Fionnbharth (A,B), Muddy (C), D29 (D) and the two-phage (E–G) and three-phage (H) cocktails infecting *M. smegmatis* at MOIs 10^{-7}–100 under planktonic growth (average ± standard deviation, n ≥ 3).

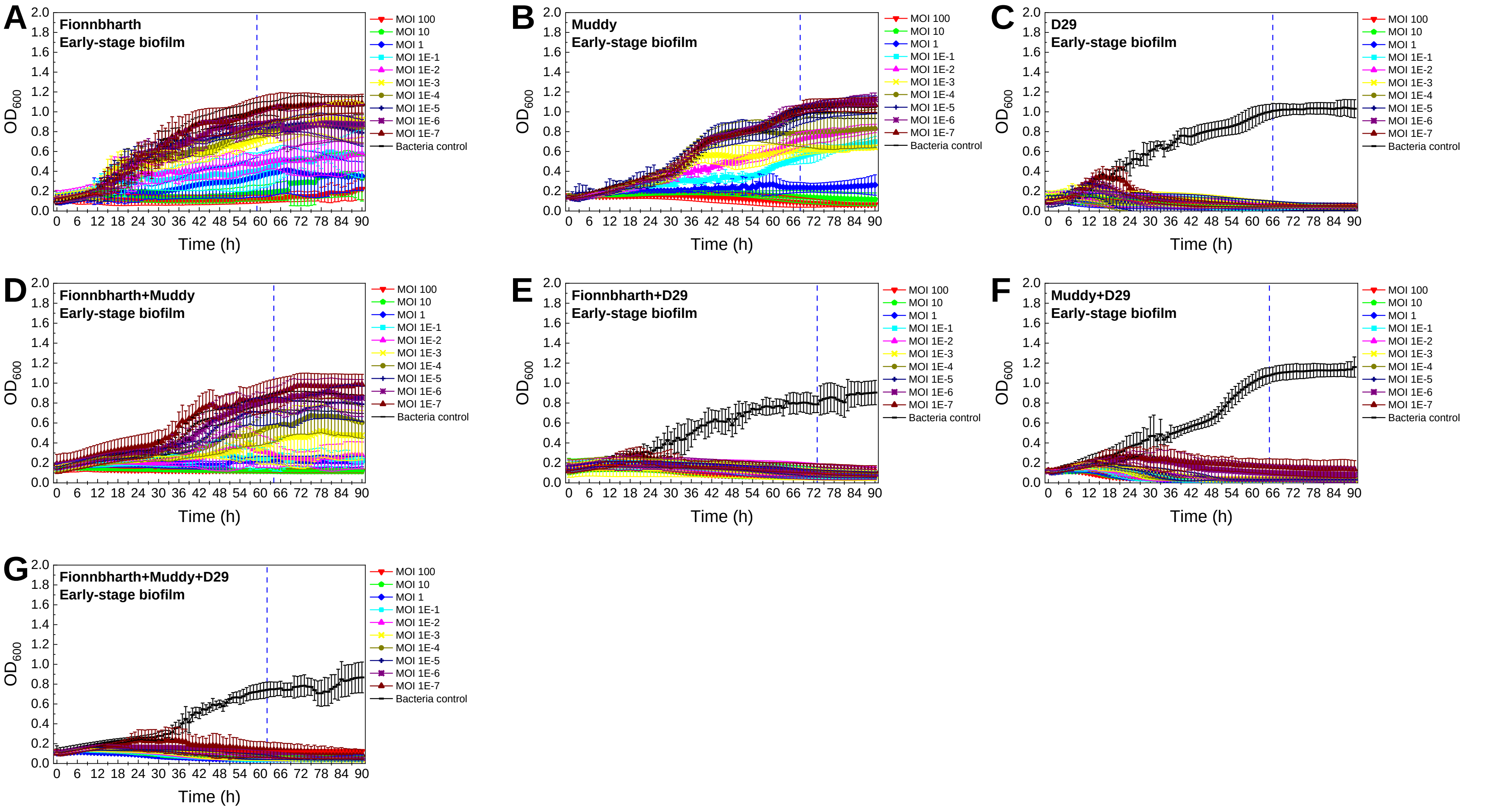

Supplementary Figure 2. Reduction curves under early-stage biofilm growth. Reduction curves for phages Fionnbharth (A), Muddy (B), D29 (C) and the two-phage (D–F) and three-phage (G) cocktails infecting *M. smegmatis* at MOIs 10^{-7}–100 under early-stage biofilm growth (average ± standard deviation, n ≥ 3).

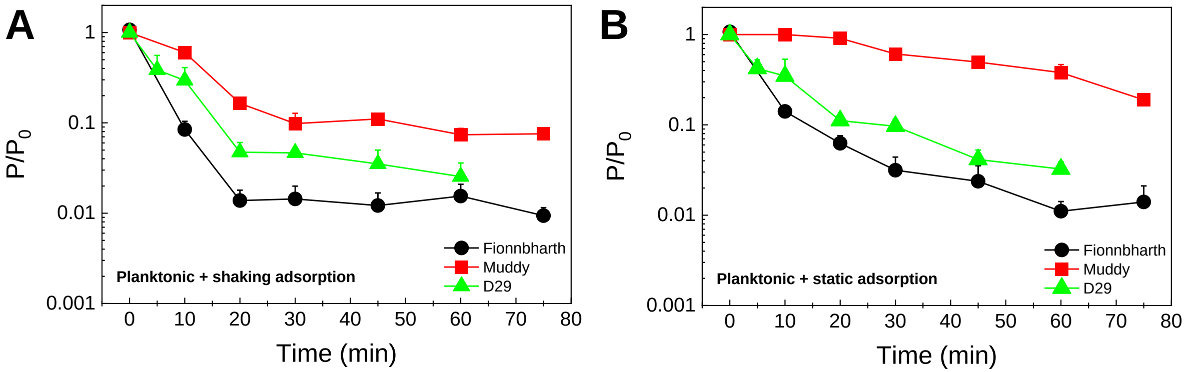

Supplementary Figure 3. Comparison of the adsorption of mycobacteriophages to planktonic *M. smegmatis*. The free phage concentration P relative to the initial phage concentration P_0 of Fionnbharth, Muddy, and D29 adsorbing to planktonic *M. smegmatis*. The adsorption was measured under shaking (A) and static (B) adsorption conditions (average ± standard deviation, n ≥ 3).

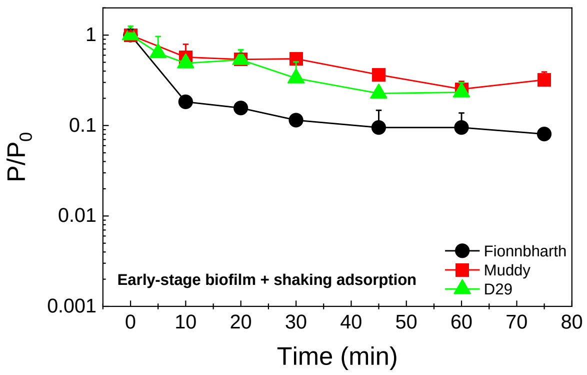

Supplementary Figure 4. Comparison of the adsorption of mycobacteriophages to early-stage biofilm of *M. smegmatis*. The free phage concentration P relative to the initial phage concentration P_0 of Fionnbharth, Muddy, and D29 adsorbing to early-stage biofilm of *M. smegmatis.* The adsorption was measured under shaking adsorption conditions (average ± standard deviation, n ≥ 3).
